# Adaptive Low-Resolution Combination Search for Reference-Independent Image Super-Resolution

**DOI:** 10.3390/s26020725

**Published:** 2026-01-21

**Authors:** Ye Tian

**Affiliations:** Key Laboratory of High-Efficiency and Clean Mechanical Manufacture (Ministry of Education), National Experimental Teaching Demonstration Center for Mechanical Engineering (Shandong University), School of Mechanical Engineering, Shandong University, Jinan 250061, China; tianyeah@mail.sdu.edu.cn

**Keywords:** image super-resolution, reference-free reconstruction, adaptive search algorithm, subpixel shift, degradation modeling

## Abstract

Accurately reconstructing high-resolution (HR) images remains challenging in scenarios where HR observations cannot be captured due to optical, hardware, or cost constraints. To address this limitation, we introduce an image super-resolution (SR) framework that reconstructs HR content solely from multiple low-resolution (LR) measurements, without relying on any HR reference images. The proposed method formulates a unified degradation model that describes how HR pixels contribute to LR observations under subpixel shifts and anisotropic downsampling. Based on this model, we develop an adaptive search algorithm capable of identifying the minimal and most informative combination of LR images required to equivalently represent the latent HR image. The selected LR images are then used to construct a solvable linear system whose solution directly yields the HR pixel values. Experiments conducted on the USAF 1951 resolution target demonstrate that the proposed approach improves Peak Signal-to-Noise Ratio (PSNR) and Structural Similarity (SSIM) by 27.33% and 44.64%, respectively, achieving a resolvable spatial frequency of 228 line pairs per millimeter. In semiconductor chip inspection, PSNR and SSIM increase by 22.36% and 40.38%. These results verify that the proposed LR-combination-based strategy provides a physically interpretable and highly practical alternative for applications in which HR reference images cannot be obtained.

## 1. Introduction

Image super-resolution (SR) plays an essential role in improving image quality and restoring intricate details, which has proven useful in numerous sectors such as medical imaging, satellite remote sensing, and biometric technology [1,2,3]. Conventional SR methods use high-resolution (HR) images as references, implementing strategies such as patch matching, texture transfer, and frequency domain analysis to refine low-resolution images. Notable progress in this area includes [4,5,6,7,8]. However, obtaining HR images is often difficult or infeasible in many practical circumstances, which considerably limits the use of SR models that require HR reference images [9,10].

Reference-free super-resolution methods have attracted increasing attention due to their ability to improve image quality without requiring access to HR images [11,12]. Mishra [13] proposed an unsupervised super-resolution framework that employs contrastive learning to extract texture features, which are subsequently refined through iterative neural network optimization to achieve super-resolved outputs. The Synthetic Multi-Orientation Resolution Enhancement algorithm was introduced by Zhao [14], which simulates paired low- and high-resolution magnetic resonance imaging data by applying synthetic degradations to native HR scans. Deepthi [15] developed a Gompertz-function convergence war accelerometric optimization generative adversarial network for hyperspectral image super-resolution in remote sensing. This approach leverages a Gompertz-function-based optimization strategy to generate HR images and uses a pre-trained Inception-v3 model to evaluate the realism and diversity of generated images based on feature distribution similarity. Ortega [16] used a controllable displacement device to capture low-resolution images at specific subpixel positions and applied a multi-image super-resolution algorithm to reconstruct a high-resolution image from four frames. Existing reference-free super-resolution methods rely on proxy metrics for quality assessment. Although these metrics show heuristic value, SR reconstruction remains an ill-posed problem where multiple HR solutions exist for one LR input. The ground-truth solution may not optimize these metrics, and excessive focus on them can produce pseudo-sharp results that distort semantic structures and morphological features [17,18]. Consequently, developing a substitute reference that truly replicates real HR imaging for physically reliable super-resolution reconstruction remains a challenge.

To address these challenges, this paper proposes a reference-free image super-resolution method based on a low-resolution image search algorithm. The method first constructs an image degradation model to characterize the relationship between the high-resolution image and its corresponding low-resolution observations. Based on this model, an adaptive search algorithm is developed to identify the optimal combination of subpixel-shifted and downsampled low-resolution images by analyzing their features and degradation characteristics. The super-resolved image is then reconstructed by solving a system of equations formulated from the selected low-resolution image combinations. Experimental results presented later in this paper demonstrate the effectiveness of the proposed method.

## 2. Methods

### 2.1. Search Algorithm for Low-Resolution Image Combination

**Figure 1:**
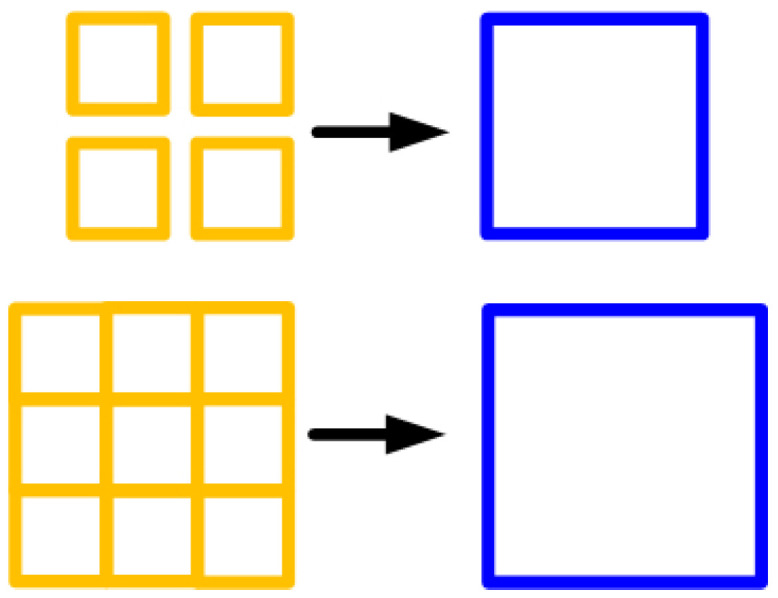
Schematic of pixel binning.

Recall that previous studies employed the pixel binning method [19,20] to improve image quality in low-light and high-noise conditions by reducing spatial resolution, as shown in Figure 1. Under the assumption that different low-resolution observations are produced by this resolution–reduction process, each low-resolution observation was represented as a linear combination of multiple high-resolution pixels. Building on this, we introduce a novel adaptive search algorithm capable of intelligently determining the optimal combination of low-resolution images, thereby providing an efficient and equivalent depiction of the original high-resolution image. Note that our degradation model follows the same principle as pixel binning, which is applicable only to digital images, and subpixel information requires high-precision motion devices.

Pixels are the fundamental units that make up a digital image. According to [21], the value of the pixel is equal to the integral of the light intensity function over its spatial area divided by that area. Ideal high-resolution pixels $hp_{ij}$ can be expressed as

(1) $$hp_{ij}=\frac{1}{l^{2}}\int_{(i-\frac{1}{2})l}^{(i+\frac{1}{2})l}\int_{(j-\frac{1}{2})l}^{(j+\frac{1}{2})l}f\left(x,y\right)\;dx\;dy.$$

Let *l* denote the edge length of the high-resolution pixel, where *i* and *j* are natural numbers that index the location of the pixel. The function f(x,y) describes the distribution of light intensity in the two-dimensional spatial domain. An ideal high-resolution image $I_{h}$, composed of mz and nz high-resolution pixels along the horizontal and vertical dimensions, respectively, can be expressed as:

(2) $$I_{h}=\frac{1}{l^{2}}\sum_{i=1}^{mz}\sum_{j=1}^{nz}\int_{(i-\frac{1}{2})l}^{(i+\frac{1}{2})l}\int_{(j-\frac{1}{2})l}^{(j+\frac{1}{2})l}f(x,y)dxdy.$$

Here, *m* and *n* are positive integers, and *z* represents the degradation factor, an integer greater than 2 by which the image is downscaled and subsequently upscaled during super-resolution. The resolution of the high-resolution image is mz × nz, occupying a sensor spatial dimension of mzl × nzl. However, in practical imaging, we cannot obtain the ideal high-resolution image but can only acquire an actual low-resolution image $I_{l}$ with a larger pixel size:

(3) $$I_{l}=\sum_{i=1}^{m}\sum_{j=1}^{n}\frac{1}{{\left(zl\right)}^{2}}\int_{0}^{zl}\int_{0}^{zl}f(x,y)dxdy.$$

The resolution of a low-resolution image is *m* × *n*, occupying a sensor area of mzl × nzl. The pixels of the actually acquired low-resolution image are denoted as lp:

(4) $$lp_{ij}=\frac{1}{{\left(zl\right)}^{2}}\int_{(i-\frac{z}{2})l}^{(i+\frac{z}{2})l}\int_{(j-\frac{z}{2})l}^{(j+\frac{z}{2})l}f(x,y)dxdy=\frac{1}{z^{2}}\sum_{1}^{z^{2}}hp_{ij}.$$

**Figure 2:**
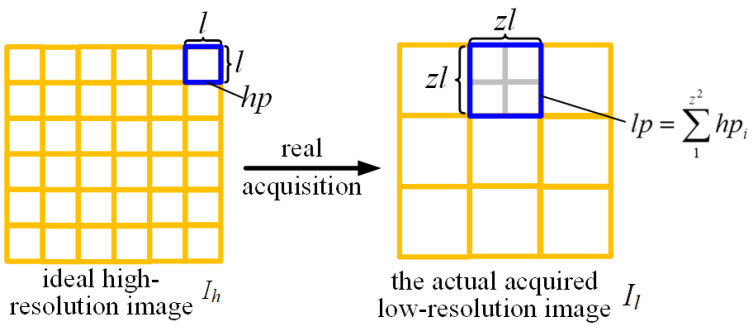
Image degradation during actual capture.

According to the theory of pixel binning, lp can be expressed as the arithmetic mean of $z^{2}$ high-resolution pixels. The relationship between the ideal high-resolution image and the actual acquired low-resolution image is illustrated in Figure 2.

Figure 2 shows the established image coordinate system, which takes the top-left corner of the image as the origin, with the positive x-direction to the right and the positive y-direction downward. Based on the acquired low-resolution image $I_{l}$, subpixel images and further downsampled pixel images can be derived through offset sampling and downsampling techniques. The subpixel image can be expressed as

(5) $$\begin{array}{c} \left\{\begin{array}{c} I_{sub}^{x}=\frac{1}{{\left(zl\right)}^{2}}\sum_{i=1}^{m}\sum_{j=1}^{n}\int_{0}^{(z+∆)l}\int_{0}^{zl}f(x,y)dxdy,\\ I_{sub}^{y}=\frac{1}{{\left(zl\right)}^{2}}\sum_{i=1}^{m}\sum_{j=1}^{n}\int_{0}^{zl}\int_{0}^{(z+∆)l}f(x,y)dxdy. \end{array}\right. \end{array}$$

A subpixel is defined as an image unit acquired by introducing an in-plane displacement, at a subpixel scale, of the imaging sensor relative to the scene. Formally, $I_{sub}^{x}$ is the image shifted by ∆x along the x-direction, and $I_{sub}^{y}$ is the image shifted by ∆y along the y-direction. The corresponding subpixel can be expressed as:

(6) $$\begin{array}{c} sp_{∆x∆y}=\frac{1}{l^{2}}\int_{(iz-\frac{z}{2}+∆x)l}^{(iz+\frac{z}{2}+∆x)l}\int_{(jz-\frac{z}{2}+∆y)l}^{(jz+\frac{z}{2}+∆y)l}f(x,y)dxdy=\frac{1}{z^{2}}\sum_{1}^{z^{2}}hp_{ij}. \end{array}$$

**Figure 3:**
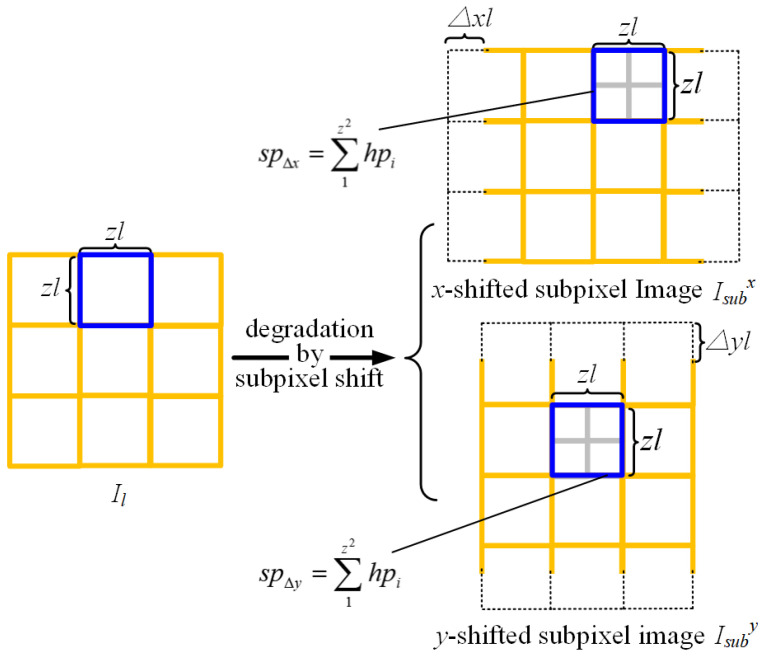
Image degradation during subpixel shift.

The subpixel can also be expressed as the arithmetic mean of $z^{2}$ high-resolution pixels. The relationship between the actual captured low-resolution images and the subpixel-shifted images is shown in Figure 3.

In addition to subpixel images, the downsampled image can be expressed as:

(7) $$\begin{array}{c} \left\{\begin{array}{c} I_{el}^{x}=\frac{1}{\lambda zl^{2}}\sum_{i=1}^{m}\sum_{j=1}^{nz/\lambda }\int_{0}^{\lambda l}\int_{0}^{zl}f(x,y)dxdy\\ I_{el}^{y}=\frac{1}{\lambda zl^{2}}\sum_{i=1}^{mz/\lambda }\sum_{j=1}^{n}\int_{0}^{zl}\int_{0}^{\lambda l}f(x,y)dxdy\\ I_{el}^{xy}=\frac{1}{{\left(\lambda l\right)}^{2}}\sum_{i=1}^{mz/\lambda }\sum_{j=1}^{nz/\lambda }\int_{0}^{\lambda l}\int_{0}^{\lambda l}f(x,y)dxdy. \end{array}\right. \end{array}$$

A downsampled pixel refers to a larger sensor pixel formed by merging multiple adjacent sensor pixels, which inherently corresponds to a lower spatial resolution. In this context, $I_{el}^{x}$ and $I_{el}^{y}$ represent the images produced by downsampling along the *x* and *y* axes, respectively, while $I_{el}^{xy}$ denotes the image downsampled along both directions. Here, λ is a positive integer that exceeds *z*, acting as the downsampling factor. The downsampled pixels can be expressed as:

(8) $$\begin{array}{c} \left\{\begin{array}{c} ep_{\lambda }^{x}=\frac{1}{\lambda zl^{2}}\int_{(i\lambda -\frac{\lambda }{2})l}^{(i\lambda +\frac{\lambda }{2})l}\int_{(jz-\frac{z}{2})l}^{(jz+\frac{z}{2})l}f(x,y)dxdy=\frac{1}{\lambda z}\sum_{1}^{\lambda z}hp_{ij}\\ ep_{\lambda }^{y}=\frac{1}{\lambda zl^{2}}\int_{(iz-\frac{z}{2})l}^{(iz+\frac{z}{2})l}\int_{(j\lambda -\frac{\lambda }{2})l}^{(j\lambda +\frac{\lambda }{2})l}f(x,y)dxdy=\frac{1}{\lambda z}\sum_{1}^{\lambda z}hp_{ij}\\ ep_{\lambda }^{xy}=\frac{1}{{\left(\lambda l\right)}^{2}}\int_{(i\lambda -\frac{\lambda }{2})l}^{(i\lambda +\frac{\lambda }{2})l}\int_{(j\lambda -\frac{\lambda }{2})l}^{(j\lambda +\frac{\lambda }{2})l}f(x,y)dxdy=\frac{1}{\lambda^{2}}\sum_{1}^{\lambda^{2}}hp_{ij} \end{array}\right. \end{array}$$

**Figure 4:**
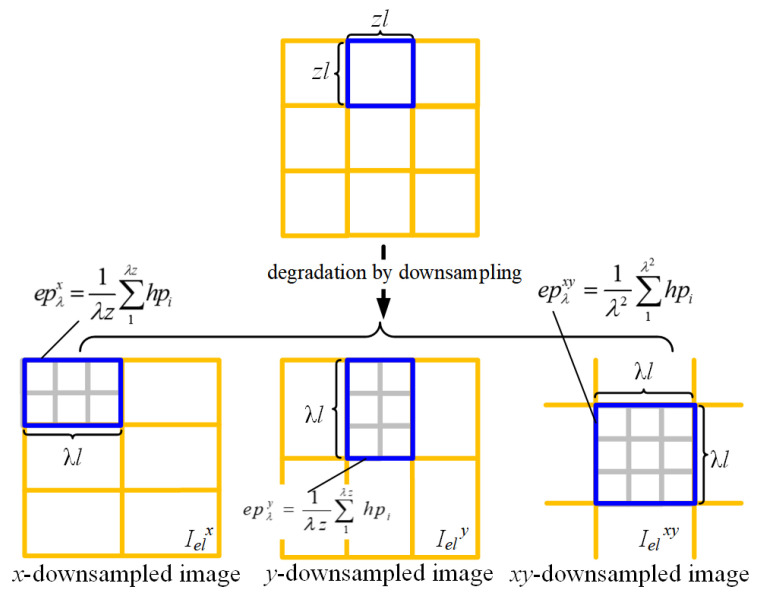
Image degradation during downsampling.

Pixels downsampled along either the x- or y-direction alone can be represented as the arithmetic mean of z·λ high-resolution pixels, while pixels downsampled in both directions correspond to the arithmetic mean of $\lambda^{2}$ high-resolution pixels. The relationship between actually captured low-resolution images and the downsampled images is shown in Figure 4.

**Figure 5:**
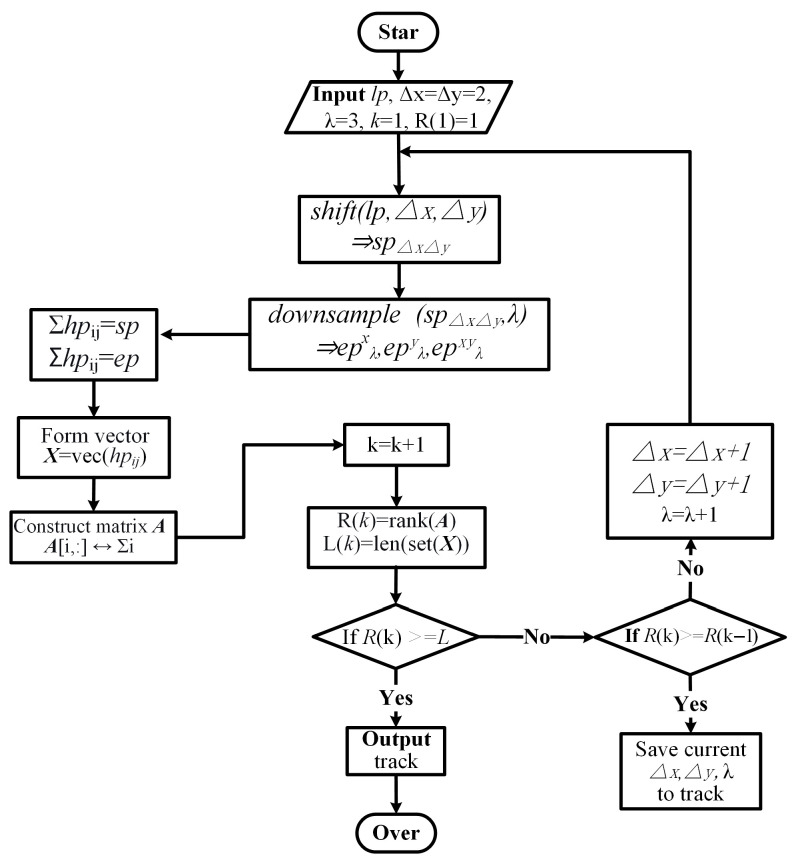
Searching for low-resolution pixel combinations.

Purchasing a high-resolution image (2) directly is not practical in real-world scenarios. However, various types of low-resolution images can be obtained efficiently. As described in (6) and (8), each low-resolution pixel can be expressed as the arithmetic average of a set of high-resolution pixels. Unlike existing studies that compute low-resolution pixels from known high-resolution pixels, our objective is to recover unknown high-resolution pixels using only the observed low-resolution measurements. A single equation derived from one low-resolution pixel is insufficient for this purpose. However, by jointly solving multiple equations that express different low-resolution pixels as linear combinations of high-resolution pixels, the rank of the resulting system can be increased, making the recovery of high-resolution pixels feasible. In this context, this study introduces a low-resolution image combination search algorithm as shown in Figure 5.

The objective of this algorithm is to search through an infinite number of low-resolution images and identify a combination that can equivalently represent a high-resolution image. According to the formula, an image is essentially a set of pixels, and there is a one-to-one correspondence between them. Therefore, in our search algorithm, pixels, rather than the entire image, are treated as the basic search unit. In this work, the adaptive search algorithm was implemented in Python 3.13 and executed on a desktop equipped with an Intel Core i5 CPU, 8 GB RAM (Intel, Santa Clara, CA, USA), and an NVIDIA GeForce GT 1030 GPU (Nvidia, Santa Clara, CA, USA), completing the computation in 1.26 s. The search algorithm proceeds through three calculation steps.

Step 1: Beginning with the low-resolution pixel lp, the process generates subpixels (sp) and downsampled pixels (ep) depending on ∆x, ∆y, and λ. Express each low-resolution pixel as a summation of high-resolution pixels, and reformulate all summation constraints into a linear system AX=B, where each row of *A* corresponds to one summation operator, and *X* denotes the vector of distinct high-resolution pixels.

Step 2: Calculate the rank of matrix *A*. If rank(A) is greater than or equal to the number of elements in *X*, the current parameters (∆x,∆y,λ) are stored and the search is stopped. Otherwise, if the rank increases compared to the previous iteration, the current parameters are recorded on the track.

Step 3: The parameters ∆x, ∆y, and λ are updated, new low-resolution pixels $s_{p}$ and $e_{p}$ are generated, and the above procedure is repeated until the stopping condition is satisfied.

According to the search algorithm, when employing a super-resolution factor of z=2, the associated sequence of low-resolution pixels is: $sp_{11}$, $sp_{21}$, $sp_{12}$, $sp_{22}$, $ep_{11}^{x}$, $ep_{11}^{y}$, $ep_{21}^{x}$, $ep_{12}^{y}$, $ep_{11}^{xy}$, as detailed in Table 1. The first and second columns list the nine low-resolution pixels that need to be collected. The third column shows their corresponding degradation parameters. Every element in this sequence is linked to a distinct low-resolution image. Consequently, reconstructing the desired high-resolution image is feasible by strategically obtaining just nine low-resolution images.

### 2.2. Simulation

**Figure 6:**
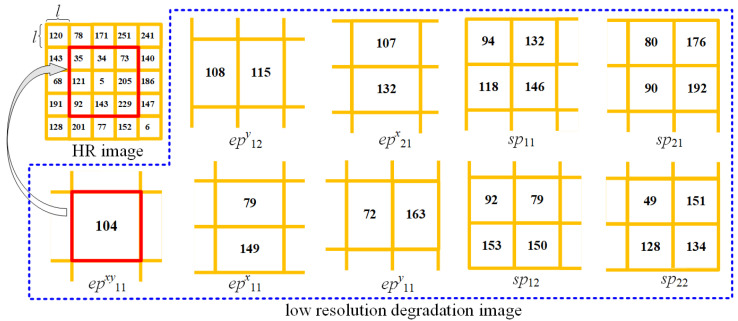
High-resolution image generation from nine retrieved low-resolution images.

To evaluate the efficacy of the proposed search method, a simulation was performed with a randomly generated high-resolution image. The image is a standard 8-bit grayscale with pixel values randomly chosen from 0 to 255. This setup provides a consistent and unbiased basis for the computational procedures, ensuring a robust assessment of the method’s general applicability. The low-resolution image, referred to as $sp_{11}$, is obtained by applying a twofold degradation to the high-resolution pixel data. According to the subpixel displacement parameter ∆ obtained by the search algorithm, other subpixel images $sp_{12}$, $sp_{21}$, and $sp_{22}$ are collected. Based on the downsampling parameter λ, the corresponding downsampled images are acquired. Both the subpixel images and downsampled images are depicted within the blue dashed boxes in Figure 6.

In Figure 6, the pixel sizes vary according to their resolutions. Pixels of higher resolutions are characterized by smaller sizes, whereas those with lower resolutions are larger. We identify the largest downsampled pixel, as marked by the red bounding box, as the basic computational unit. Each computational unit corresponds to nine high-resolution pixels, denoted $x_{1}$ to $x_{9}$, organized in row-major order from top left to bottom right. Consequently, we can establish a system of linear equations represented by AX=B. In this formulation, A represents the coefficient matrix, X is the vector containing the unknown variables, and B is the vector of constants. The explicit form of the coefficient matrix is provided:

(9) $$A=\left[\begin{array}{ccccccccc} 1 & 1 & 1 & 1 & 1 & 1 & 1 & 1 & 1\\ 0 & 0 & 0 & 0 & 1 & 1 & 0 & 1 & 1\\ 0 & 0 & 0 & 1 & 1 & 0 & 1 & 1 & 0\\ 0 & 1 & 1 & 0 & 1 & 1 & 0 & 0 & 0\\ 1 & 1 & 0 & 1 & 1 & 0 & 0 & 0 & 0\\ 0 & 1 & 1 & 0 & 1 & 1 & 0 & 1 & 1\\ 0 & 0 & 0 & 1 & 1 & 1 & 1 & 1 & 1\\ 1 & 1 & 1 & 1 & 1 & 1 & 0 & 0 & 0\\ 1 & 1 & 0 & 1 & 1 & 0 & 1 & 1 & 0 \end{array}\right]$$

The representation of the vector of unknown variables is:

(10) $$X={\left(x_{1},x_{2},x_{3},x_{4},x_{5},x_{6},x_{7},x_{8},x_{9}\right)}^{T}$$

The expression of the vector of constant terms is shown in (11).

(11) $$\begin{array}{c} B=(104\times 9146\times 4,90\times 4,79\times 4,49\times 4,\\ \;\;\;\;\;\;\;115\times 6132\times 6,79\times 6,72\times 6{)}^{T} \end{array}$$

The elements of the constant vector are derived by multiplying each low-resolution pixel value by a scaling factor. This factor is determined by the ratio of the area of the low-resolution pixel to that of the high-resolution pixel. Then we can obtain the solution:

(12) $$\left(\begin{array}{ccc} x_{1} & x_{2} & x_{3}\\ x_{4} & x_{5} & x_{6}\\ x_{7} & x_{8} & x_{9} \end{array}\right)=\left(\begin{array}{ccc} 38 & 34 & 72\\ 120 & 4 & 206\\ 88 & 148 & 229 \end{array}\right)$$

For convenience, we rearrange $x_{1}$ through $x_{9}$ into a matrix here. It should be noted that these results were obtained using only the low-resolution images (Figure 6) as input, whereas the provided high-resolution images were not involved in the reconstruction process.

To quantitatively evaluate the super-resolution results, we compare the reconstructed image (corresponding to $x_{1}$ through $x_{9}$) with the reference region marked by the red bounding box in Figure 6. The evaluation metrics are the Peak Signal-to-Noise Ratio (PSNR) and the Structural Similarity (SSIM), which are widely adopted in image super-resolution research. The PSNR calculation first requires computing the Mean Squared Error (MSE) between the reference image *R* and the reconstructed image *R*, where a×b denotes the total number of pixels in the images and can be adaptively adjusted according to the input image size:

(13) $$\mathrm{MSE}\left(R,D\right)=\frac{1}{ab}\sum_{i=1}^{a}\sum_{j=1}^{b}{\left[R(i,j)-D(i,j)\right]}^{2}.$$

For 8-bit images with the maximum pixel intensity L=255, the PSNR (in decibels) is then derived as:

(14) $$\mathrm{PSNR}\left(R,D\right)=10\log_{10}\left(\frac{L^{2}}{\mathrm{MSE}(R,D)}\right).$$

Higher PSNR values indicate better reconstruction fidelity. On the other hand, the SSIM metric evaluates perceptual quality through three comparative components: luminance (*l*), contrast (*c*), and structure (*s*):

(15) $$\left\{\begin{array}{c} l\left(R,D\right)=\frac{2\mu_{R}\mu_{D}+C_{1}}{\mu_{R}^{2}+\mu_{D}^{2}+C_{1}}\\ c\left(R,D\right)=\frac{2\sigma_{R}\sigma_{D}+C_{2}}{\sigma_{R}^{2}+\sigma_{D}^{2}+C_{2}}\\ s\left(R,D\right)=\frac{\sigma_{RD}+C_{3}}{\sigma_{R}\sigma_{D}+C_{3}}. \end{array}\right.$$

where μ, σ denote local means and standard deviations, respectively, $\sigma_{RD}$ is the cross-covariance, and $C_{i}$ are stability constants. The composite SSIM index combines these components:

(16) $$\mathrm{SSIM}\left(R,D\right)={\left[l\left(R,D\right)\right]}^{\alpha }\cdot {\left[c\left(R,D\right)\right]}^{\beta }\cdot {\left[s\left(R,D\right)\right]}^{\gamma }.$$

Following standard practice [22], we set α=β=γ=1 and $C_{3}=C_{2}/2$, with $C_{1}={\left(0.01L\right)}^{2}$, $C_{2}={\left(0.03L\right)}^{2}$. In this validation case study, the evaluation metrics PSNR and SSIM yield values of 39.6798 dB and 0.9991, respectively. According to the criteria established in [23], these results confirm that high-quality reconstruction can be achieved solely from low-resolution inputs without relying on high-resolution references. These results further substantiate the precision of our innovative low-resolution image combination search algorithm, which efficiently identifies an optimal subset from a theoretically infinite pool of potential low-resolution image combinations to accurately approximate the target high-resolution image. Note that although the simulation experiment demonstrates only a single case, the pixel values of the test image can be arbitrarily replaced. To further ensure the robustness of the conclusions, we provide a theoretical validation in the Appendix A that does not rely on specific pixel values.

## 3. Experiment and Results

### 3.1. Experimental Setup

**Figure 7:**
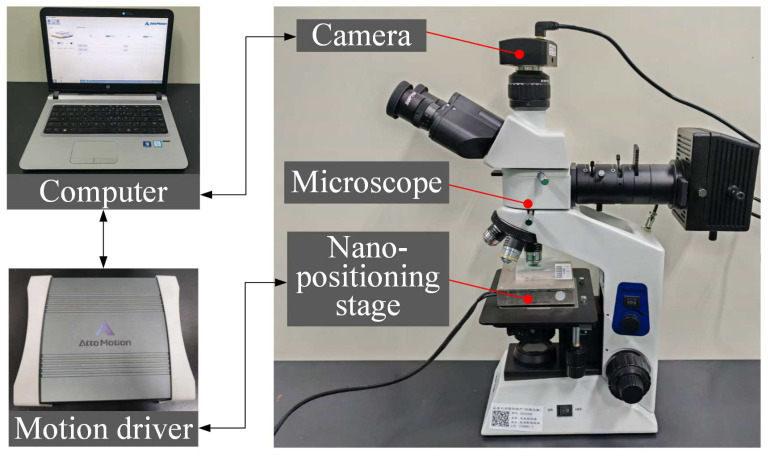
Experiment setup.

An experimental setup was constructed, as illustrated in Figure 7, to validate the effectiveness of the proposed reference-free image super-resolution based on the low-resolution image search algorithm. The setup comprised a microscope equipped with interchangeable objective lenses (20×, 50×, and 100×), a digital camera, a motion driver, a computer, and a nano-positioning stage. The digital camera, mounted on the eyepiece port of the microscope, was used to capture image sequences. Both the camera and the motion driver were connected to the computer that was responsible for generating motion control signals and coordinating image acquisition.

The model and manufacturer of all equipment are detailed in Table 2.

The nano-positioning stage serves as the core actuating mechanism for subpixel-shifted image generation, with its key performance specifications summarized in Table 3. Driven by piezoelectric ceramics, this stage enables precise nanometer-level displacement of the observed samples, thereby providing a reliable motion control foundation for high-accuracy acquisition of subpixel-shifted images.

The microscope enables image acquisition at different magnifications (50× and 100×). Throughout our experiments, every type of low-resolution image was obtained using a magnification of 50×. Separately, high-resolution ground truth images were captured at 100× magnification and used solely for quantitative evaluation of reconstruction results. It is important to note that the 100× reference images were used exclusively as the benchmark for evaluation purposes and did not participate in the reconstruction process.

The experiments consist of two parts: resolution detection using a calibration target and surface inspection of a semiconductor chip. For both experiments, the procedures were identical. Initially, under the 50× objective lens, the first image captured after focusing was used as the base image corresponding to (3). Then, the low-resolution image search algorithm was executed to generate acquisition positions. These positions were used to drive the nano-positioning stage accordingly, acquiring additional low-resolution images through image stretching and downsampling operations. Subsequently, a system of equations such as AX=B was formulated, and the target HR pixels were obtained by solving these equations. Finally, these HR pixels were combined to reconstruct the super-resolution image. To establish comprehensive performance benchmarks, additional reference-free methods were also implemented as control experiments, including GAN-based reconstruction [15], a multiimage super-resolution (MISR) method [16], and classical interpolation algorithms [24]. These comparison methods were deliberately selected because they operate in reference-free settings and exploit multiple low-resolution images with subpixel shifts. In contrast, many recent reference-free SR approaches rely on single-image inputs or learned priors, which are not directly comparable to our acquisition model.

### 3.2. Results

**Figure 8:**
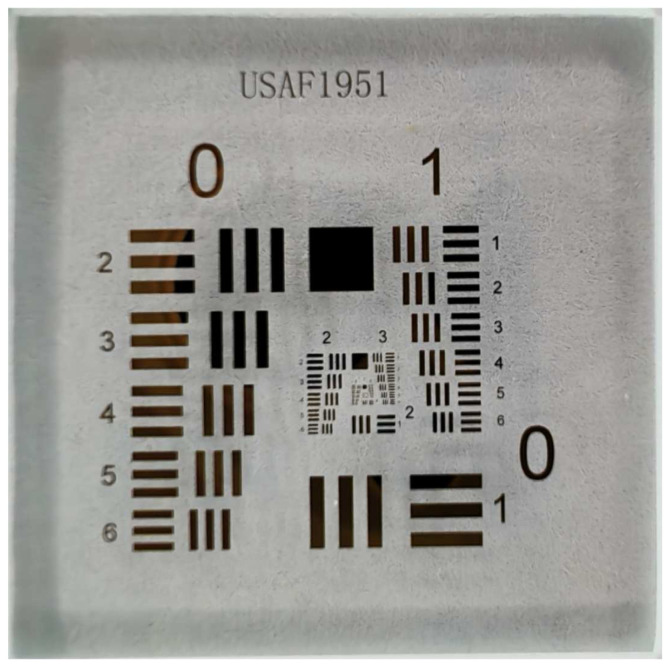
Photo of the USAF 1951 resolution test target.

The resolution performance was evaluated using the USAF 1951 resolution target, as shown in Figure 8. The target consists of multiple groups of line-pair patterns with gradually decreasing spacing, providing a precise and standardized reference for resolution assessment. Owing to its well-defined geometric progression and high manufacturing accuracy, the USAF target enables reliable benchmarking of the proposed method.

**Figure 9:**
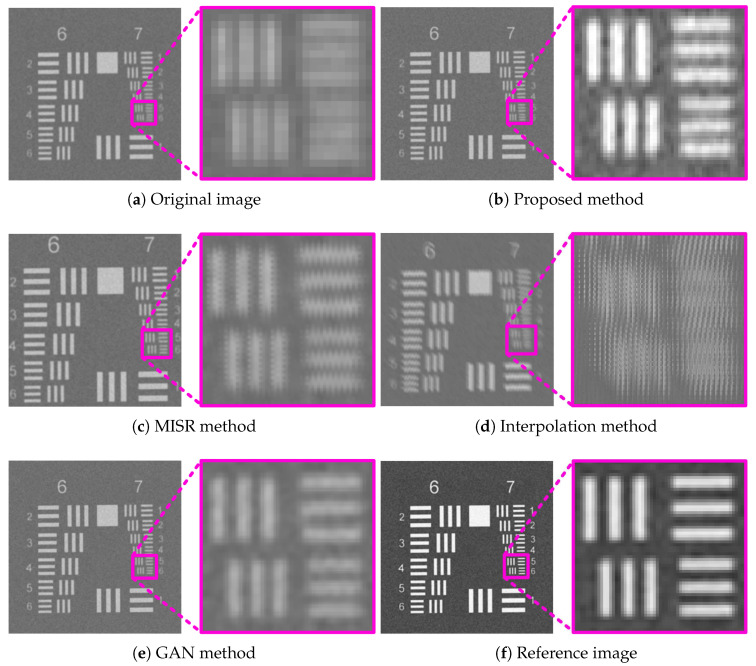
Experimental results of the USAF 1951 target.

Figure 9 presents the super-resolution results of the USAF target. To facilitate visual comparison, the region corresponding to Group 7, Element 6—containing the finest line pairs—was selected as the region of interest (ROI) and magnified. The proposed method, the GAN-based method, and the MISR method successfully resolve the finest line-pair structure corresponding to 228 lp/mm. In contrast, the interpolation-based method suffers from significant blurring after magnification and fails to recover the line-pair details, while the MISR method introduces noticeable jagged artifacts along edges despite resolving the structure.

**Figure 10:**
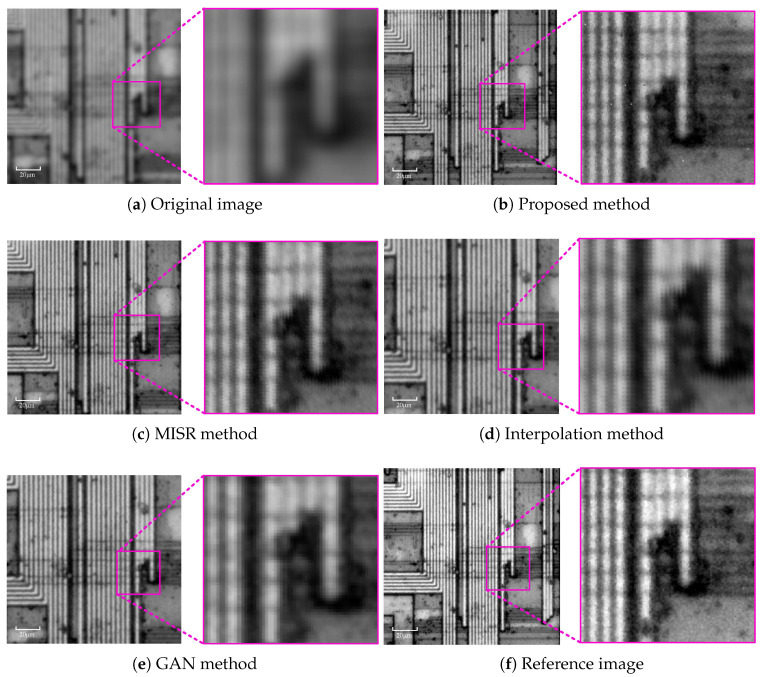
Experimental result of semiconductor chip.

The second experiment evaluates the method in the context of semiconductor surface inspection. The test sample is a BIWIN DDR4-2666 chip, a widely used high-performance memory component. Optical microscopy is commonly employed for chip inspection due to its non-contact and fast imaging characteristics, despite inherent resolution limitations. The imaging results obtained by different methods are shown in Figure 10.

In addition to the visual results, quantitative evaluations were performed using Peak Signal-to-Noise Ratio (PSNR) and Structural Similarity (SSIM) metric to measure reconstruction accuracy. The metrics for all methods in both experiments are summarized in Table 4. Here, the original image refers to the low-resolution image directly captured under the 50× objective and used as input prior to super-resolution, the baseline of interpolation corresponds to classical bicubic interpolation.

## 4. Discussion

Across both the USAF 1951 target and DDR4 chip experiments, the proposed method consistently outperforms existing approaches in terms of reconstruction quality. Compared with the best-performing baseline, PSNR increases by 27.33% and 28.10%, while SSIM increases by 44.64% and 50.98% for the two experiments, respectively. These improvements demonstrate that the proposed method not only enhances numerical accuracy but also preserves structural information more effectively.

Notably, the improvement in SSIM is more pronounced than that in PSNR. This difference can be attributed to the high-frequency quantization errors introduced during the estimation of high-resolution pixels. Since SSIM focuses on structural similarity and perceptual fidelity, it is less sensitive to such high-frequency noise. In contrast, PSNR directly reflects pixel-wise errors and therefore decreases more easily when quantization artifacts appear. This observation highlights that the proposed method yields perceptually faithful reconstructions even in the presence of minor high-frequency deviations. Overall, both subjective visual assessment and objective metrics demonstrate that the method achieves superior reconstruction performance in resolving fine structures and recovering surface details, validating its effectiveness in microscopy imaging and semiconductor inspection applications.

## 5. Conclusions

This paper presents a novel reference-free image super-resolution method based on a low-resolution image combination search algorithm. By constructing a unified degradation model that encompasses subpixel-shifted images, downsampled images, and high-resolution counterparts, a linear combination-based optimization strategy is developed to effectively search and integrate low-resolution observations. This enables accurate representation and reconstruction in the absence of high-resolution references. The experimental results validate the effectiveness of the proposed method in different test scenarios. In the USAF 1951 resolution target experiment, the proposed method improved PSNR and SSIM by 27. 33% and 44. 64%, respectively, achieving a resolution of 228 pairs of lines per millimeter. Similarly, in the DDR4 chip imaging experiment, PSNR and SSIM increased by 22.36% and 40.38%, respectively. These results demonstrate the method’s ability to address the critical challenge of missing high-resolution priors in unsupervised super-resolution reconstruction.

## Figures and Tables

**Table 1:** Search result.

No.	Search Result	Pixel (Pre-Degradation)
1	$ep_{11}^{xy}$	∆x=1, ∆y=1, λ=3
2	$sp_{12}$	∆x=0, ∆y=1, λ=0
3	$sp_{22}$	∆x=1, ∆y=1, λ=0
4	$sp_{21}$	∆x=1, ∆y=0, λ=0
5	$sp_{11}$	∆x=0, ∆y=0, λ=0
6	$ep_{21}^{x}$	∆x=1, ∆y=1, λ=3
7	$ep_{11}^{y}$	∆x=0, ∆y=0, λ=3
8	$ep_{12}^{y}$	∆x=0, ∆y=1, λ=3
9	$ep_{11}^{x}$	∆x=0, ∆y=0, λ=3

**Table 2:** Experimental setup specifications.

Device	Model	Manufacturer
camera	DH-GV400	Daheng, China
computer	EliteBook 1040	HP Inc., USA
microscope	MX4R	Soptop, Ningbo, China
nano-positioning stage	NP-XY-100	Attomotion, Rizhao, China
driver	APS-203	Attomotion, Rizhao, China

**Table 3:** Parameters of the nano-positioning stage.

Property	Value
Motion axes	2
Sensor type	Capacitive
Actuator	Piezoelectric ceramic
Travel range (closed-loop)	100 × 100 μm
Resolution (closed-loop)	0.35 nm
Repeatability	±1 nm
Resonant frequency (unloaded)	550 Hz
Maximum payload	1 kg

**Table 4:** Quantitative evaluation of experimental results.

Sample	Method	Metrics	
		PSNR	SSIM
USAF 1951 target	Original image	18.56	0.32
	Proposed method	**32.24**	**0.81**
	MISR method	25.32	0.56
	Interpolation method	19.19	0.33
	GAN method	24.90	0.54
DDR4 chip	Original image	17.42	0.32
	Proposed method	**30.87**	**0.77**
	MISR method	24.10	0.51
	Interpolation method	18.32	0.31
	GAN method	23.74	0.49

**Figure A1:**
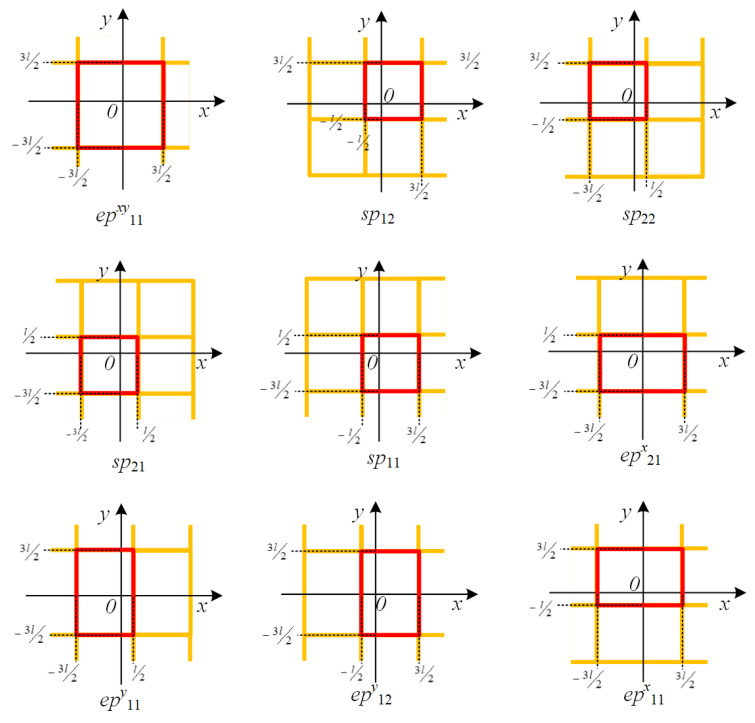
Low-resolution degradation image.

## Data Availability

The data presented in this study are available within the article (including all figures and simulation parameters). No new external datasets were generated.

## Appendix A

This appendix shows how to get small high-resolution pixels. We use nine types of large low-resolution pixels found by search. We do not assign amplitude values to the pixels. We compute them step by step. Based on Figure 6 in Section 2.2, we set up a coordinate system for the nine types of low-resolution pixels, as shown in Figure A1. Then we get their math formula:

(A1)
$$\begin{array}{cc} 9ep_{11}^{xy} & =\frac{1}{l^{2}}\int_{x_{c}-\frac{3}{2}l}^{x_{c}+\frac{3}{2}l}\int_{y_{c}-\frac{3}{2}l}^{y_{c}+\frac{3}{2}l}f(x,y)dxdy,\\ 4sp_{12} & =\frac{1}{l^{2}}\int_{x_{c}-\frac{1}{2}l}^{x_{c}+\frac{3}{2}l}\int_{y_{c}-\frac{3}{2}l}^{y_{c}+\frac{1}{2}l}f(x,y)dxdy,\\ 4sp_{22} & =\frac{1}{l^{2}}\int_{x_{c}-\frac{3}{2}l}^{x_{c}+\frac{1}{2}l}\int_{y_{c}-\frac{1}{2}l}^{y_{c}+\frac{3}{2}l}f(x,y)dxdy,\\ 4sp_{21} & =\frac{1}{l^{2}}\int_{x_{c}-\frac{3}{2}l}^{x_{c}+\frac{1}{2}l}\int_{y_{c}-\frac{3}{2}l}^{y_{c}+\frac{1}{2}l}f(x,y)dxdy,\\ 4sp_{11} & =\frac{1}{l^{2}}\int_{x_{c}-\frac{1}{2}l}^{x_{c}+\frac{3}{2}l}\int_{y_{c}-\frac{3}{2}l}^{y_{c}+\frac{1}{2}l}f(x,y)dxdy,\\ 6ep_{21}^{x} & =\frac{1}{l^{2}}\int_{x_{c}-\frac{3}{2}l}^{x_{c}+\frac{3}{2}l}\int_{y_{c}-\frac{3}{2}l}^{y_{c}+\frac{1}{2}l}f(x,y)dxdy,\\ 6ep_{11}^{y} & =\frac{1}{l^{2}}\int_{x_{c}-\frac{3}{2}l}^{x_{c}+\frac{1}{2}l}\int_{y_{c}-\frac{3}{2}l}^{y_{c}+\frac{3}{2}l}f(x,y)dxdy,\\ 6ep_{12}^{y} & =\frac{1}{l^{2}}\int_{x_{c}-\frac{1}{2}l}^{x_{c}+\frac{3}{2}l}\int_{y_{c}-\frac{3}{2}l}^{y_{c}+\frac{3}{2}l}f(x,y)dxdy,\\ 6ep_{11}^{x} & =\frac{1}{l^{2}}\int_{x_{c}-\frac{3}{2}l}^{x_{c}+\frac{3}{2}l}\int_{y_{c}-\frac{1}{2}l}^{y_{c}+\frac{3}{2}l}f(x,y)dxdy. \end{array}$$

In (A1), we use the origin point $0(x_{c},y_{c})$ as a reference to describe the integral terms of the pixels. The normalization coefficient before integration is set to $\frac{1}{l^{2}}$. The goal of image super-resolution is to obtain nine pixels with side length *l*. We define these target high-resolution pixels as $hp_{1}$ to $hp_{9}$. To get these high-resolution pixels equally, we do the following linear calculation on the nine types of low-resolution pixels. This gives the initial value for the iteration.

(A2) $$\begin{array}{cc} hp_{1}^{1} & ={9ep}_{11}^{xy}-4sp_{12}-4sp_{21}+4sp_{11},\\ hp_{2}^{1} & =4sp_{12},\\ hp_{3}^{1} & =9ep_{11}^{xy}-4sp_{22},\\ hp_{4}^{1} & =4sp_{21}-4sp_{11},\\ hp_{5}^{1} & =4sp_{11},\\ hp_{6}^{1} & =6ep_{21}^{x}-4sp_{21},\\ hp_{7}^{1} & =6ep_{11}^{y}-9ep_{11}^{xy},\\ hp_{8}^{1} & =6ep_{12}^{y}-4sp_{12},\\ hp_{9}^{1} & =6ep_{11}^{x}-9ep_{11}^{xy}. \end{array}$$

In the initial iteration, the high-resolution pixel hp has its subscript indicating the pixel number and its superscript representing the iteration count. From this result, we obtain the following:

(A3) $$\begin{array}{cc} hp_{1}^{2} & =hp_{1}^{1}-hp_{8}^{1},\\ hp_{2}^{2} & =hp_{2}^{1}-hp_{3}^{1}-hp_{5}^{1}-hp_{6}^{1},\\ hp_{3}^{2} & =hp_{3}^{1}-hp_{6}^{1},\\ hp_{4}^{2} & =hp_{4}^{1}+hp_{4}^{6},\\ hp_{5}^{2} & =hp_{5}^{1}-hp_{6}^{1}-hp_{8}^{1},\\ hp_{6}^{2} & =hp_{6}^{1},\\ hp_{7}^{2} & =hp_{7}^{1}+hp_{3}^{1},\\ hp_{8}^{2} & =hp_{8}^{1},\\ hp_{9}^{2} & =hp_{9}^{1}. \end{array}$$

In the second iteration, we used a selective update strategy: $hp_{6}$, $hp_{8}$, and $hp_{9}$ kept their previous values and were not updated. To make it simpler, these unchanged pixels will not be shown in later steps. Their values will stay the same automatically. Using this method, we get the results for the third iteration.

(A4) $$\begin{array}{cc} hp_{1}^{3} & =hp_{1}^{2}-hp_{6}^{2}=\frac{1}{l^{2}}\int_{x_{c}-\frac{3}{2}l}^{x_{c}-\frac{1}{2}l}\int_{y_{c}+\frac{1}{2}l}^{y_{c}+\frac{3}{2}l}f\left(x,y\right)\;dx\;dy,\\ hp_{2}^{3} & =hp_{2}^{2}+hp_{7}^{1}+hp_{8}^{1}=\frac{1}{l^{2}}\int_{x_{c}-\frac{1}{2}l}^{x_{c}+\frac{1}{2}l}\int_{y_{c}+\frac{1}{2}l}^{y_{c}+\frac{3}{2}l}f\left(x,y\right)\;dx\;dy,\\ hp_{3}^{3} & =hp_{3}^{2}-hp_{7}^{2}=\frac{1}{l^{2}}\int_{x_{c}+\frac{1}{2}l}^{x_{c}+\frac{3}{2}l}\int_{y_{c}+\frac{1}{2}l}^{y_{c}+\frac{3}{2}l}f\left(x,y\right)\;dx\;dy,\\ hp_{4}^{3} & =hp_{4}^{2}-hp_{7}^{2}+hp_{8}^{2},\\ hp_{7}^{3} & =hp_{7}^{2}-hp_{8}^{2},\\ hp_{9}^{3} & =hp_{9}^{2}+hp_{7}^{2}. \end{array}$$

During the third iteration, $hp_{1}$, $hp_{2}$, and $hp_{3}$ have converged to the target size $l^{2}$, matching the expected high-resolution pixels. They are therefore set as the final solution. The remaining pixels, which have not yet met this convergence condition, will continue to be updated in later iterations.

(A5) $$\begin{array}{cc} hp_{4}^{4} & =hp_{4}^{3}+hp_{9}^{3}=\frac{1}{l^{2}}\int_{x_{c}-\frac{3}{2}l}^{x_{c}-\frac{1}{2}l}\int_{y_{c}-\frac{1}{2}l}^{y_{c}+\frac{1}{2}l}f\left(x,y\right)\;dx\;dy,\\ hp_{5}^{4} & =hp_{5}^{3}-hp_{9}^{3}=\frac{1}{l^{2}}\int_{x_{c}-\frac{1}{2}l}^{x_{c}+\frac{1}{2}l}\int_{y_{c}-\frac{1}{2}l}^{y_{c}+\frac{1}{2}l}f\left(x,y\right)\;dx\;dy,\\ hp_{6}^{4} & =hp_{6}^{3}+hp_{9}^{3}=\frac{1}{l^{2}}\int_{x_{c}+\frac{1}{2}l}^{x_{c}+\frac{3}{2}l}\int_{y_{c}-\frac{1}{2}l}^{y_{c}+\frac{1}{2}l}f\left(x,y\right)\;dx\;dy,\\ hp_{7}^{4} & =hp_{7}^{3}-hp_{9}^{3}=\frac{1}{l^{2}}\int_{x_{c}-\frac{3}{2}l}^{x_{c}-\frac{1}{2}l}\int_{y_{c}-\frac{3}{2}l}^{y_{c}-\frac{1}{2}l}f\left(x,y\right)\;dx\;dy,\\ hp_{8}^{4} & =hp_{8}^{3}+hp_{9}^{3}=\frac{1}{l^{2}}\int_{x_{c}-\frac{1}{2}l}^{x_{c}+\frac{1}{2}l}\int_{y_{c}-\frac{3}{2}l}^{y_{c}-\frac{1}{2}l}f\left(x,y\right)\;dx\;dy,\\ hp_{9}^{4} & =-hp_{9}^{3}=\frac{1}{l^{2}}\int_{x_{c}+\frac{1}{2}l}^{x_{c}+\frac{3}{2}l}\int_{y_{c}-\frac{3}{2}l}^{y_{c}-\frac{1}{2}l}f\left(x,y\right)\;dx\;dy. \end{array}$$

By combining (A4) and (A5), we can obtain the final converged values for $hp_{1}$ to $hp_{9}$. The input of the whole iteration process is the 9 low-resolution pixels we found, and the output is 9 high-resolution pixels. All high-resolution pixels meet the convergence condition of having an integration area equal to $l^{2}$. Their integration areas do not overlap in space and, together, they fully cover the smallest common integration region. A pixel is the smallest unit of an image. Repetition of this process can obtain all the pixels of the target high-resolution image.
